# Efficient Microwave Assisted Syntheses of 2,5-Diketopiperazines in Aqueous Media ^†^

**DOI:** 10.3390/molecules14082836

**Published:** 2009-07-31

**Authors:** Lemuel Pérez-Picaso, Jaime Escalante, Horacio F. Olivo, María Yolanda Rios

**Affiliations:** 1 Centro de Investigaciones Químicas, Universidad Autónoma del Estado de Morelos, Avenida Universidad 1001, Col. Chamilpa, 62209 Cuernavaca, Morelos, México; 2 Medicinal and Natural Products Chemistry, The University of Iowa, Iowa City, IA 52242, USA

**Keywords:** 2,5-diketopiperazines, DKPs, cyclic dipeptides, microwave irradiation

## Abstract

Aqueous *in situ* one-pot *N*-Boc-deprotection-cyclization of *Nα*-Boc-dipeptidyl-*tert*-butyl and methyl esters under microwave irradiation afforded 2,5-diketopiperazines (DKPs) in excellent yields. This protocol is rapid, safe, environmentally friendly, and highly efficient, and showed that the *tert*-butoxy moiety is also an excellent leaving group for these cyclizations.

## 1. Introduction

Cyclic peptides are a very important family of bioactive compounds easily available both from natural sources (plants, animals or microorganisms) or by means of synthetic methods. Their chemical properties, like the lack of charges at the amine and carboxylic terminal groups and the lack of zwitterionic character, confer to these molecules high lipophilicity [1], and fast membrane absorption in the digestive tract because of their high permeability [2]. The structural rigidity of the cyclic peptides increases its affinity and selectivity toward protein ligands, making their half-lives *in vivo* much greater than those of linear peptides [3]. Among them, 2,5-diketopiperazines (DKPs, also known as cyclic dipeptides, 2,5-dioxopiperazines, cyclo(dipeptides), or anhydride dipeptides) are the smallest cyclopeptides. These peptides are most commonly found as natural products [4], showing antimicrobial [5], antitumoral and antiviral [6], cytotoxic [7], and neuroprotective effects [8], among other activities. Some DKPs are stable to proteolysis (enzymatic degradation), an important feature for their high activity. All these properties make DKPs an interesting group of molecules for the development of new therapeutic agents.

The DKP core derives, chemically and biosynthetically, from folding and head-tail cyclization between *N* and *C*-terminal amino acids of linear dipeptides [9]. These heterocyclic compounds possess two amide groups (with acceptor-donor properties) with the possibility of including up to four hydrogen bonds [10]. DKPs can be synthesized in solution or in solid phase from commercially available and appropriately protected chiral α-amino acids. Their syntheses can be carried out with the appropriate linear dipeptide [11,12,13], followed by *N*-deprotection and cyclization using either basic (aminolysis of dipeptide ester in methanolic ammonia, Fischer method) [10,14], neutral (aminolysis of dipeptide ester in methanol, autoaminolysis) [15], or acidic conditions (Suzuki method) [16]. Some of these methods variously result in good reaction yields [17], low reaction yields [18], with or without epimerization [19].

Nowadays, microwave heating can be used to obtain good results from previously unsuccessful or low-yielding reactions. Under microwave heating conditions the reaction times can be reduced from days to hours, from hours to minutes, or from minutes to seconds, and the reaction yields can be greatly increased [20,21]. DKPs syntheses using microwave assistance has barely been investigated. Some examples of the use of this methodology are the syntheses of dimeric structures based on intermolecular DKP formation by activation of C-terminal glycine monomers [22], the solvent-free synthesis of DKPs in one-pot deprotection-cyclization of *N*-Boc-dipeptidyl ethyl and methyl esters [23], and the DKPs formation using dipeptide methyl ester hydrochlorides in water in three [24] and two steps [25]. In the present study, we report the syntheses of DKPs using *Nα*-Boc-dipeptidyl methyl and *tert*-butyl esters in water under microwave irradiation. DKPs were obtained in excellent yields without epimerization employing this general and highly efficient protocol.

## 2. Results and Discussion

**Figure 1:**
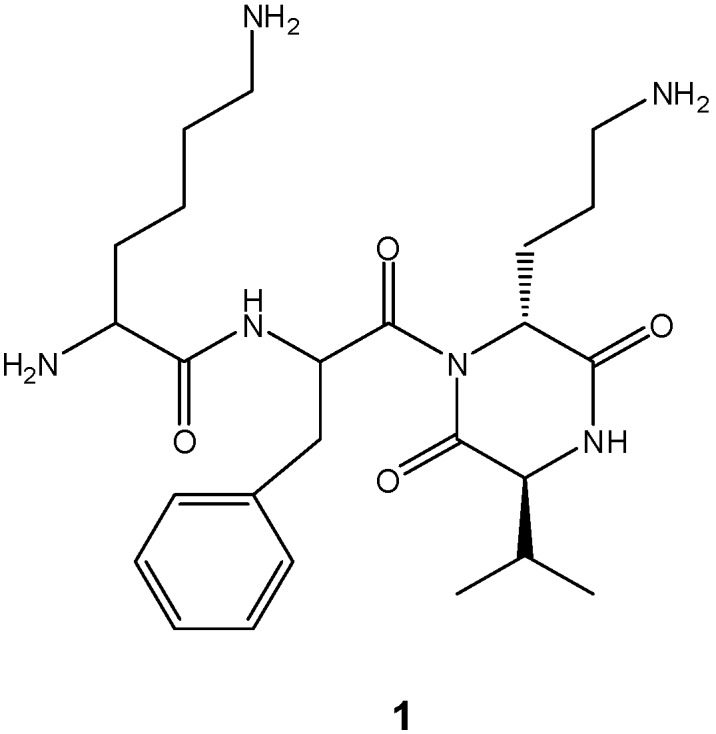
Structure of cyclo[*N*-(Lys-Phe)-Orn-Val] (**1**).

The formation of DKPs occurs through an intramolecular aminolysis depending largely on the nature and the sequence of the amino acids in solution [11]. Under these conditions, when DKP formation is an undesired reaction, *tert-*butyl ester protection for the carboxy terminus function is used to prevent ring closure of dipeptides under basic conditions, because it provides a poor leaving group and steric hindrance. Nucleophilic removal of this protecting group with ring formation has been observed only in a few cases [26,27]. We studied the cyclization reaction assisted by microwave heating with the purpose of investigating the possible use of *Nα*-Boc-dipeptidyl *tert*-butyl esters as DKPs precursors, as part of a synthetic strategy toward the *cis*-DKP fragment of the natural product cyclo[*N*-(Lys-Phe)-Orn-Val] (1, Figure 1) [28]. We report our findings herein.

In an effort to explore optimized conditions for the cyclization under microwave irradiation, Boc-Orn(Cbz)-Val-O*t*Bu (**2a**) was subjected to different reaction variables, such as solvent, temperature (T), irradiation power (W), and exposure time exchange (Table 1). Toluene, toluene-isopropanol (1:1) mixture, and xylene were unsuccessful in the transformation of dipeptide **2a** to DKP **3a** (entries 1-6). When DMF was used as solvent at 200 °C, 300 W, and 5 min reaction time, DKP **3a** was obtained in 61% yield (entry 7). Water resulted a better solvent for the cyclization (entries 8-11). As can be observed in Table 1, with water as solvent, 200 °C, 300 W, and 5 min reaction time resulted in the best conditions, yielding **3a** in 89% yield. However, pressure in the sealed reaction tube increased dramatically due to the generation of CO_2_ gas, increasing the risk of breaking the reaction vessel. Increasing the temperature and the reaction time did not improve the reaction yield any further (entry 11). The optimized conditions were then used in all cyclizations of *Nα*-Boc-dipeptidyl esters **2a-2l** to DKPs **3a-3k**.

**Table 1:** Cyclization of dipeptide **2a** to diketopiperazine **3a** under microwave irradiation.^a^

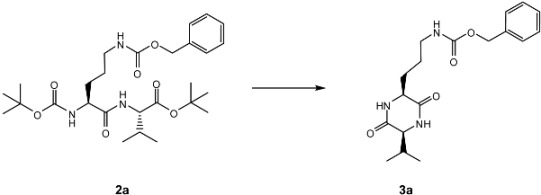					
**Entry**	**Solvent**	**T (°C)**	**Power (W)**	**Time (min)**	**3a (% yield)**
**1**	toluene	170	160	10	nr
**2**		170	180	10	nr
**3**		200	250	10	nr
**4**		200	300	10	nr
**5**	toluene-isopropanol (1:1)	200	150	10	nr
**6**	xylene	200	300	10	nr
**7**	DMF	200	300	5	61
**8**	H_2_O	200	250	1	22
**9**		200	250	2.5	85
**10**		200	300	5	89
**11**		250	250	10	86

nr = no reaction; ^a^ Reactions performed in a monomode microwave CEM Discover apparatus.

DKPs **3a-3k** were synthesized in two steps: dipeptide formation (starting with *Nα*-Boc-terminal and C-O*t*Bu or C-OMe amino acids, to obtain the corresponding *Nα*-Boc-dipeptidyl esters **2a-2l**) and subsequent cyclization (Table 2).

**Table 2:** Coupling of amino acids and ring closure under microwave irradiation.^a^

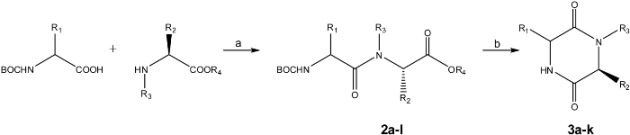								
(a) EDAC, HOBt, DMAP, TEA/CH_2_Cl_2_, 5 °C, then overnight at rt; (b) H_2_O (1 mL), MW (250 °C, 250 W and 150 psi) for 10 min.								
**Entry**	**R_1_**	**R_2_**	**R_3_**	**R_4_**	**Compound**	**Yield (%)**	**Compound**	**Yield (%)**
**1**	(*S*)-Propyl-*N*H-Cbz	Isopropyl	H	O*t*Bu	**2a**	94	**3a**	86^b^
**2**	(*R*)-Propyl-*N*H-Cbz	Isopropyl	H	O*t*Bu	**2b**	98	**3b**	99^b^
**3**	Propyl-*N*H-Cbz	Benzyl	H	O*t*Bu	**2c**	91	**3c**	95^b^
**4**	Propyl-*N*H-Cbz	Benzyl	H	OMe	**2d**	77	**3c**	99^b^
**5**	H	Benzyl	H	O*t*Bu	**2e**	98	**3e**	99^b^
**6**	Benzyl	Benzyl	H	O*t*Bu	**2f**	98	**3f**	96^b^
**7**	Isopropyl	Benzyl	H	O*t*Bu	**2g**	99	**3g**	99^b^
**8**	H	Isopropyl	H	O*t*Bu	**2h**	73	**3h**	93^b^
**9**	Benzyl	Isopropyl	H	O*t*Bu	**2i**	80	**3g**	73^b^
**10**	Isopropyl	Isopropyl	H	O*t*Bu	**2j**	83	**3j**	99^b^
**11**	Benzyl	H	CH_3_	O*t*Bu	**2k**	85	**3k**	84^c^
**12**	Benzyl	H	CH_3_	OMe	**2l**	82	**3k**	99^c^

^a^ Reactions performed in a monomode microwave CEM Discover apparatus; ^b^ Crude yield (pure by NMR); ^c^ Isolated yield after chromatography .

Data in Table 2 clearly shows that the cyclization step assisted by microwave heating yielded DKP compounds **3a-3k** in excellent yields. Because *N*-Boc protecting groups are unstable at temperatures higher than 90 °C, microwave irradiation should deprotect the amines facilitating the spontaneous intramolecular aminolysis [29]. Nature and size of alkyl group (R_1_) on Cα of *Nα*-Boc amino acid residue have no effect on the course and reaction yield when the C terminus amino acid is phenylalanine (entries 3-7). However, they do influence the cyclization yield when the amino acid at C terminus is valine (entries 1, 8-10). When valine is present, the amino acid residue sequence is important on the cyclization yield (entries 7 and 9) because *β*-branched amino acid at C terminus (Cα isopropyl group) exerts a bulky effect on ring closure, diminishing the reaction yield (99 vs 73%). Entries 1 and 3 where a benzyl group replaced an isopropyl one corroborate this steric effect as the reaction yield diminished from 95 to 86%. The leaving groups O*t*Bu and OMe (ester group nature, entries 3,4 and 11,12) have no effect on course and reaction yield, as corroborated by the excellent yields obtained for compounds **3a-3h** and **3j-3k**. Optical rotation values of compounds **3a-3k** and their comparison with literature data showed that cyclization reaction proceeded without Cα chiral center epimerization of the amino acid residues. The cyclization of different *N*α-Boc-dipeptidyl esters (even O*t-*Bu) in water assisted by microwaves is a rapid, secure, environmentally friendly and highly efficient method to produce *cis*-DKPs with high optical purity. This reaction conditions are compatible with the presence of Cbz protecting groups. This protocol was used to obtain the *trans*-DKP fragment **3b** of compound **1**, which was synthesized in quantitative yield starting of Boc-D-Orn(Cbz)-Val-O*t*Bu (**2b**, entry 2).

The structures of compounds **2a-2l** and **3a-3k** were established on the basis of the analysis of their spectroscopic data. For compounds **2a-2l**, carbamate, amide, and ester groups showed IR absorptions at 3,364-3,283, 2,979-2,972 and 1,690-1,665, and 1,744-1,712 cm^-1^, respectively; Carbonyl amide, ester, *N*-Boc-carbamate, and *Nδ*-Cbz-carbamate groups showed ^13^C-NMR resonance signals at δ 172.35-169.07, δ 172.10-167.89, δ 157.09-155.07, and δ 156.98-156.94. Carbamate and amide groups in compounds **3a-3k** produce IR absorptions at 3,440-3,426, and 2,977-2,925 and 1,678-1,664 cm^-1^, respectively. Amide groups showed ^13^C-NMR resonance signals at δ 167.83-166.00, and *Nδ*-Cbz-carbamate carbonyls give resonance signals in the δ 156.06-155.79 range.

## 3. Experimental

### 3.1. General procedures

Reactions were performed in sealed vessels in a monomode microwave CEM Discover apparatus and the temperature was evaluated by infrared. Melting points were obtained in a Fisher Johns melting point apparatus and are uncorrected. Infrared (IR) spectra were obtained in KBr on a Bruker Vector 22 IR spectrometer. Optical rotations were measured with sodium light (unless otherwise specified) on a Perkin-Elmer 341 MC polarimeter. ^1^H- and ^13^C-NMR spectra were recorded on a Varian Unity 400 spectrometer at 400 MHz for ^1^H-NMR, ^1^H-^1^H COSY, HSQC, and HMBC, and at 100 MHz for ^13^C- NMR, using CDCl_3_ or DMSO as solvents, as indicated. Chemical shifts are reported in ppm (δ) relative to the TMS signal. FAB^+^MS, and HRFAB^+^MS were recorded on a JEOL JMStation-JM 700 mass spectrometer at 70 eV in a matrix of glycerol. Flash column chromatography (FCC) and analytical thin-layer chromatography (TLC) were performed using silica gel 230-400 mesh and pre-coated silica gel 60 F254 Merck plates, respectively. Boc-Phe-OH, Boc-Val-OH, Boc-Orn(Cbz)-OH, Boc-Gly-OH, Sar-OMe, Sar-O*t*Bu, Phe-OMe, Phe-O*t*Bu, Val-O*t*Bu, EDAC, TEA, and DMAP were obtained from Aldrich, HOBt was obtained from ANASPEC, and all chemicals were used without further purification.

### 3.2. General procedure for the syntheses of dipeptides ***2a-2j***

A mixture of Boc-amino acid (1 mmol), amino ester hydrochloride (1 mmol), EDAC (1.5 mmol), HOBt (1 mmol) and DMAP (0.1 mmol) were dissolved in dry CH_2_Cl_2_ (10 mL). Mixture was cooled to 5 °C and then TEA (1 mmol) was added. Reaction was stirred at 5 °C for further 30 min, then allowed to warm up to room temperature and stirred overnight. Reaction mixture was treated with sat. NH_4_Cl soln. (20 mL). The organic phase was separated and the aqueous layer was extracted with CH_2_Cl_2_ (3 × 15 mL). Combined organic layers were washed with brine (2 × 15 mL) and with water (2 ×15 mL) and dried over Na_2_SO_4_. Solvent was removed under vacuum. The residue was purified by FCC.

### 3.3. General procedure for the syntheses of dipeptides ***2k-2l***

A mixture of Boc-amino acid (2.4 mmol), amino ester hydrochloride (2 mmol), EDAC (2.4 mmol), HOBt (2.4 mmol) and DMAP (0.1 mmol) were dissolved in dry CH_2_Cl_2_ (5 mL). Mixture was cooled to 5 °C and then TEA (2.4 mmol) was added. Reaction was stirred at 5 °C for further 30 min, then allowed to warm up to room temperature and stirred for two days. Reaction mixture was treated with sat. NH_4_Cl soln. (20 mL). The organic phase was separated and the aqueous layer was extracted with CH_2_Cl_2_ (3 × 15 mL). Combined organic layers were washed with brine (2 × 15 mL) and with water (2 × 15 mL) and dried over Na_2_SO_4_. Solvent was removed under vacuum. The residue was purified by FCC.

*Boc-Orn(Cbz)-Val-OtBu* (**2a**): Colorless syrup; [α] + 8.5 (c 1.09, CHCl_3_); IR: 3,336, 2,973, 2,935, 2,878, 1,712, 1,666, 1,532, 1,454, 1,368, 1,254, 1,162, 1,018 cm^-1^; ^1^H-NMR (CDCl_3_) δ 7.28-7.20 (5H, m, Ar), 6.85 (1H, bs, NH-Val), 5.29 (1H, bs, NH-Orn), 5.18 (1H, bs, NHδ-Orn), 5.04 (1H, d, *J* = 12.4 Hz, CH_2_-Cbz), 5.01 (1H, d, *J* = 12.4 Hz, CH_2_-Cbz), 4.33 (1H, dd, *J* = 9.2, 4.8 Hz, Hα-Val), 4.24 (1H, bs, Hα-Orn), 3.29 (1H, bs, Hδ-Orn), 3.08 (1H, bd, *J* = 13.2 Hz, Hδ’-Orn), 2.09 (1H, dh, *J* = 9.2, 7.2 Hz, Hβ-Val), 1.78 (1H, m, Hβ-Orn), 1.51 (3H, m, Hβ’-Orn, Hγ-Orn), 1.37 (9H, s, CH_3_Boc), 1.35 (9H, s, CH_3_*t*Bu), 0.86 (3H, d, *J* = 7.2 Hz, Hγ-Val), 0.84 (3H, d, *J* = 7.2 Hz, Hγ’-Val); ^13^C-NMR (CDCl_3_) δ 172.26 (s, CO-Orn), 170.84 (s, CO-Val), 156.97 (s, CO-Cbz), 155.79 (s, CO-Boc), 136.63 (s, Ar), 128.49 (d, Ar), 128.11 (d, Ar), 128.07 (d, Ar), 81.87 (s, C-Boc), 79.89 (s, C-*t*Bu), 66.80 (t, CH_2_-Cbz), 57.65 (d, Cα-Val), 53.41 (d, Cα-Orn), 40.02 (t, Cδ-Orn), 31.28 (d, Cβ-Val), 30.10 (t, Cβ-Orn), 28.50 and 28.20 (q, CH_3_-Boc, CH_3_-*t*Bu), 26.35 (t, Cγ-Orn), 19.17 (q, Cγ-Val), 17.25 (q, Cγ’-Val); FAB^+^MS *m/z*: 522 (53) [M + H]^+^, 466 (11) [M + H - C_4_H_8_]^+^, 422 (13) [M + H - C_5_H_8_O_2_]^+^, 414 (15) [M - C_7_H_7_O]^+^, 366 (100) [M + H - C_5_H_8_O_2_ - C_4_H_8_]^+^, 258 (13), 213 (20), 91 (100) [C_7_H_7_]^+^, 57 (44) [C_4_H_9_]^+^; HRFAB^+^MS: observed 522.3176 [M + H]^+^, (calcd. for C_27_H_44_N_3_O_7_, 522.3179).

*Boc-D-Orn(Cbz)-Val-OtBu* (**2b**): Colorless syrup; [α] + 15.7 (c 1.04, CHCl_3_); IR: 3,339, 2,973, 2,935, 2,877, 1,712, 1,666, 1,525, 1,456, 1,391, 1,368, 1,254, 1,165, 1,022, 738, 699 cm^-1^; ^1^H-NMR (CDCl_3_) δ 7.36-7.25 (5H, m, Ar), 6.89 (1H, bs, NH-Val), 5.36 (1H, bd, *J* = 6.6 Hz, NH-Orn), 5.25 (1H, bs, NHδ-Orn), 5.08 (2H, s, CH_2_-Cbz), 4.39 (1H, dd, *J* = 8.8, 4.4 Hz, Hα-Val), 4.23 (1H, bs, Hα-Orn), 3.21 (2H, m, Hδ-Orn), 2.10 (1H, dh, *J* = 9.2, 7.2 Hz, Hβ-Val), 1.85 (1H, m, Hβ-Orn), 1.60 (3H, m, Hβ’-Orn, Hγ-Orn), 1.45 (9H, s, CH_3_Boc), 1.43 (9H, s, CH_3_*t*Bu), 0.92 (3H, d, *J* = 7.0 Hz, Hγ-Val), 0.89 (3H, d, *J* = 7.0 Hz, Hγ’-Val); ^13^C-NMR (CDCl_3_) δ 172.06 (s, CO-Orn), 170.78 (s, CO-Val), 156.74 (s, CO-Cbz), 155.71 (s, CO-Boc), 136.69 (s, Ar), 128.50 (d, Ar), 128.06 (d, Ar), 82.00 (s, C-Boc), 80.06 (s, C-*t*Bu), 66.68 (t, CH_2_-Cbz), 57.60 (d, Cα-Val), 54.09 (d, Cα-Orn), 40.37 (t, Cδ-Orn), 31.46 (d, Cβ-Val), 30.07 (t, Cβ-Orn), 28.47 and 28.19 (q, CH_3_-*t*Bu, CH_3_-*t*Bu), 26.39 (t, Cγ-Orn), 19.11 (q, Cγ-Val), 17.77 (q, Cγ’-Val); FAB^+^MS *m/z*: 522 (9) [M + H]^+^, 466 (3) [M + H - C_4_H_8_]^+^, 422 (4) [M + H - C_5_H_8_O_2_]^+^, 410 (9), 366 (39) [M + H - C_9_H_17_O_2_]^+^, 258 (8), 213 (15), 91 (100) [C_7_H_7_]^+^, 72 (28) [C_4_H_8_O]^ +^, 57 (38) [C_4_H_9_]^+^; HRFAB^+^MS: observed 522.3176 [M + H]^+^, (calcd. for C_27_H_44_N_3_O_7_, 522.3179).

*Boc-Orn(Cbz)-Phe-OtBu* (**2c**): White solid; Mp 104-105 °C {lit [30] 102 °C}; [α] + 25.8 (c 1.01, CHCl_3_); IR: 3,364, 2,977, 2,880, 1,732, 1,690, 1,668, 1,524, 1,452, 1,368, 1,280, 1,240, 1,164, 1,027 cm^-1^; ^1^H-NMR (CDCl_3_): δ 7.35-7.15 (10H, m, Ar), 6.82 (1H, d, *J* = 6.8 Hz, NH-Phe), 5.18 (1H, d, *J* = 6.8 Hz, NH-Orn), 5.06 (2H, d, *J* = 12.8 Hz, CH_2_-Cbz, NHδ-Orn), 5.02 (1H, d, *J* = 12.8 Hz, CH_2_-Cbz), 4.70 (1H, dd, *J* = 14.0, 6.0 Hz, Hα-Phe), 4.24 (1H, bs, Hα-Orn), 3.33 (1H, bs, Hδ-Orn), 3.13 (1H, m, Hδ’-Orn), 3.08 (1H, dd, *J* = 13.6, 14.0 Hz, Hβ-Phe), 3.04 (1H, dd, *J* = 13.6, 6.0 Hz, Hβ’-Phe), 2.14 (1H, m, Hγ-Orn), 1.81 (1H, m, Hγ’-Orn), 1.53 (2H, m, Hβ, Hβ’-Orn), 1.43 (9H, s, CH_3_*t*Bu), 1.37 (9H, s, CH_3_Boc); ^13^C-NMR (CDCl_3_): δ 171.80 (s, CO-Orn), 170.48 (s, CO-Phe), 156.94 (s, CO-Cbz), 155.66 (s, CO-Boc), 136.61 (s, Ar-Cbz), 136.23 (s, Ar), 129.58 (d, Ar), 128.58 (d, Ar), 128.49 (d, Ar), 128.16 (d, Ar), 127.03 (d, Ar), 82.41 (s, C-Boc), 80.07 (s, C-*t*Bu), 66.88 (t, CH_2_-Cbz), 53.81 (d, Cα-Phe), 53.35 (d, Cα-Orn), 39.97 (t, Cδ-Orn), 38.16 (t, Cβ-Phe), 30.25 (t, Cγ-Orn), 28.44 and 28.04 (q, CH_3_-*t*Bu, CH_3_-*t*Bu), 26.27 (t, Cβ-Orn); FAB^+^MS *m/z*: 570 (56) [M + H]^+^, 556 (9) [M + H - CH_2_]^+^, 514 (8) [M + H - C_4_H_8_]^+^, 470 (56) [M + H - C_5_H_8_O_2_]^+^, 414 (90) [M + H - C_5_H_8_O_2_ - C_4_H_8_]^+^, 306 (12) [M + H - C_14_H_19_NO_3_ - CH_3_]^+^, 261 (17), 204 (14), 154 (27), 120 (27), 91 (100) [C_7_H_7_]^+^, 57 (44) [C_4_H_9_]^+^; HRFAB^+^MS: observed 570.3152 [M + H]^+^, (calcd. for C_31_H_44_N_3_O_7_, 570.3179).

*Boc-Orn(Cbz)-Phe-OMe* (**2d**): White solid; Mp 118-120 °C {lit [31] 106-118 °C}; [α] - 7.5 (c 1.01, MeOH) {lit [31] - 7.6 (c 1.1, MeOH)}; IR: 3,339, 2,974, 2,939, 2,876, 1,743, 1,677, 1,531, 1,449, 1,369, 1,278, 1,249, 1,172, 1,032 cm^-1^; ^1^H-NMR (CDCl_3_) δ 7.36-7.10 (10H, m, Ar), 7.00 (1H, d, *J* = 7.6 Hz, NH-Phe), 5.24 (1H, d, *J* = 8.0 Hz, NH-Orn), 5.12 (1H, t, *J* = 6.0 Hz, NHδ-Orn), 5.03 (1H, d, *J* = 12.8 Hz, CH_2_-Cbz), 4.99 (1H, d, *J* = 12.8 Hz, CH_2_-Cbz), 4.83 (1H, dd, *J* = 13.2, 6.4 Hz, Hα-Phe), 4.25 (1H, bs, Hα-Orn), 3.67 (3H, s, OCH_3_), 3.33 (1H, m, Hδ-Orn), 3.12 (1H, m, Hδ’-Orn), 3.11 (1H, dd, *J* = 13.6, 5.6 Hz, Hβ-Phe), 3.05 (1H, dd, *J* = 13.6, 6.8 Hz, Hβ’-Phe), 1.78 (1H, m, Hγ-Orn), 1.52 (3H, m, Hβ, Hβ’, Hγ-Orn), 1.42 (9H, s, CH_3_Boc); ^13^C-NMR (CDCl_3_) δ 172.10 (s, CO-Phe), 171.91 (s, CO-Orn), 156.98 (s, CO-Cbz), 155.68 (s, CO-Boc), 136.58 (s, Ar-Cbz), 135.95 (s, Ar), 129.31 (d, Ar), 128.64 (d, Ar), 128.55 (d, Ar), 128.14 (d, Ar), 127.14 (d, Ar), 80.04 (s, C-Boc), 66.82 (t, CH_2_-Cbz), 53.48 (d, Cα-Phe), 53.28 (d, Cα-Orn), 52.47 (q, OCH_3_), 39.95 (t, Cδ-Orn), 38.05 (t, Cβ-Phe), 30.26 (t, Cγ-Orn), 28.52 (q, CH_3_Boc), 26.24 (t, Cβ-Orn); FAB^+^MS *m/z*: 528 (11) [M + H]^+^, 472 (7) [M + H - C_4_H_9_]^+^, 428 (62) [M + H - C_5_H_8_O_2_]^+^, 320 (9) [C_17_H_24_N_2_O_4_]^+^, 275(10), 180 (19) [C_10_H_14_NO_2_]^+^, 120 (31) [C_8_H_10_N]^+^, 91 (100) [C_7_H_7_]^+^, 57 (44) [C_4_H_9_]^+^; HRFAB^+^MS: observed 528.2686 [M + H]^+^, (calcd. for C_28_H_38_N_3_O_7_, 528.2710).

*Boc-Gly-Phe-OtBu* (**2e**): Colorless syrup; [α] + 47.2 (c 1.1, CHCl_3_); IR: 3,414, 3,336, 2,979, 2,934, 1,727, 1,671, 1,521, 1,452, 1,369, 1,220, 1,160, 1,044, 942, 850, 743, 701 cm^-1^; ^1^H-NMR (CDCl_3_): δ 7.30-7.13 (5H, m, Ar), 6.72 (1H, d, *J* = 8.0 Hz, NH-Phe), 5.32 (1H, dd, *J* = 4.8, 4.8 Hz, NH-Gly), 4.75 (1H, dd, *J* = 14.0, 6.0 Hz, Hα-Phe), 3.83 (1H, dd, *J* = 16.4, 5.2 Hz, Hα-Gly), 3.74 (1H, dd, *J* = 16.4, 5.2 Hz, Hα’-Gly), 3.08 (2H, d, *J* = 6.0 Hz, Hβ-Phe), 1.44 (9H, s, CH_3_-*t*Bu), 1.39 (9H, s, CH_3_-Boc); ^13^C-NMR (CDCl_3_) δ 170.39 (s, CO-Phe), 169.07 (s, CO-Gly), 155.97 (s, CO-*t*Bu), 136.63 (s, Ar), 129.54 (d, Ar), 128.43 (d, Ar), 127.00 (d, Ar), 82.54 (s, C-Boc), 80.21 (s, C-*t*Bu), 53.66 (d, Cα-Phe), 44.32 (t, Cα-Gly), 38.22 (t, Cβ-Phe), 28.47 and 28.09 (q, CH_3_-*t*Bu, CH_3_-*t*Bu); FAB^+^MS *m/z*: 379 (41) [M + H]^+^, 323 (16) [M + H - C_4_H_8_]^+^, 267 (100) [M + H - C_4_H_8_ - C_4_H_8_]^+^, 223 (25) [M + H - C_7_H_10_NO_3_]^+^, 166 (13), 154 (57), 120 (28), 57 (38) [C_4_H_9_]^+^; HRFAB^+^MS: observed 379.2267 [M + H]^+^, (calcd. for C_20_H_31_N_2_O_5_, 379.2233).

*Boc-Phe-Phe-OtBu* (**2f**): Colorless crystals; Mp 125-127 °C; [α] + 34.1 (c 1.0, CHCl_3_); IR, ^1^H-NMR and ^13^C-NMR (CDCl_3_) are in agreement with previously reported data [32]; FAB^+^MS *m/z*: 469 (27) [M + H]^+^, 413 (10) [M + H - C_4_H_8_]^+^, 357 (58) [M + H - C_4_H_8_ - C_4_H_8_]^+^, 313 (78) [M + H - C_4_H_8_ - C_5_H_8_O_2_]^+^, 166 (15), 120 (100), 57 (45) [C_4_H_9_]^+^; HRFAB^+^MS: observed 469.2724 [M + H]^+^, (calcd. for C_27_H_37_N_2_O_5_, 469.2702).

*Boc-Val-Phe-OtBu* (**2g**): Colorless crystals; Mp 112-115 °C; [α] + 29.0 (c 1.01, CHCl_3_); IR: 3,337, 3,282, 2,972, 2,936, 2,874, 1,733, 1,689, 1,659, 1,525, 1,457, 1,371, 1,248, 1,162, 1,020, 848, 752, 696 cm^-1^; ^1^H-NMR (CDCl_3_): δ 7.30-7.15 (5H, m, Ar), 6.50 (1H, d, *J* = 7.2 Hz, NH-Phe), 5.16 (1H, d, *J* = 8.8 Hz, NH-Val), 4.74 (1H, dd, *J* = 14.0, 6.4 Hz, Hα-Phe), 3.94 (1H, dd, *J* = 8.4, 6.8Hz, Hα-Val), 3.08 (2H, dd, *J* = 6.4, 4.4 Hz, Hβ-Phe), 2.09 (1H, m, Hβ-Val), 1.45 (9H, s, CH_3_-*t*Bu), 1.38 (9H, s, CH_3_-Boc), 0.93 (3H, d, *J* = 6.8 Hz, Hγ-Val), 0.88 (3H, d, *J* = 6.4 Hz, Hγ’-Val); ^13^C-NMR (CDCl_3_) δ 171.16 (s, CO-Val), 170.46 (s, CO-Phe), 155.79 (s, CO-Boc), 136.10 (s, Ar), 129.57 (d, Ar), 128.45 (d, Ar), 127.02 (d, C_4_), 82.42 (s, C-Boc), 79.89 (s, C-*t*Bu), 60.03 (d, Cα-Val), 53.78 (d, Cα-Phe), 38.34 (t, Cβ-Phe), 31.16 (d, Cβ-Val), 28.52 and 28.09 (q, CH_3_-*t*Bu, CH_3_-Boc), 19.44 (q, Cγ-Val), 17.97 (q, Cγ’-Val); FAB^+^MS *m/z*: 421 (59) [M + H]^+^, 365 (17) [M + H - C_4_H_8_]^+^, 309 (100) [M + H - C_4_H_8_ - C_4_H_8_]^+^, 265 (95) [M + H - C_4_H_8_ - C_5_H_8_O_2_]^+^, 166 (30), 120 (60)**,** 72 (53), 57 (48) [C_4_H_9_]^+^; HRFAB^+^MS: observed 421.2666 [M + H]^+^, (calcd. for C_23_H_37_N_2_O_5_, 421.2702).

*Boc-Gly-Val-OtBu* (**2h**): Colorless oil; [α] + 23.8 (c 1.02, CHCl_3_); IR: 3,333, 2,975, 2,935, 2,878, 1,726, 1,671, 1,524, 1,458, 1,391, 1,369, 1,281, 1,251, 1,166, 1,052, 943, 848, 786 cm^-1^; ^1^H-NMR (CDCl_3_) δ 6.80 (1H, bs, NH-Val), 5.48 (1H, bd, *J* = 5.6 Hz, NH-Gly), 4.46 (1H, dd, *J* = 9.2, 4.4 Hz, Hα-Val), 3.87 (1H, dd, *J* = 16.4, 5.6 Hz, Hα-Gly), 3.80 (1H, dd, *J* = 16.4, 5.6 Hz, Hα’-Gly), 2.17 (1H, hd, *J* = 7.2, 4.4 Hβ-Val), 1.47 (9H, s, CH_3_Boc), 1.46 (9H, s, CH_3_*t*Bu), 0.94 (3H, d, *J* = 7.2 Hz, Hγ-Val), 0.89 (3H, d, *J* = 7.2 Hz, Hγ’-Val); ^13^C-NMR (CDCl_3_) δ 170.95 (s, CO-Val), 169.52 (s, CO-Gly), 156.12 (s, CO-Boc), 82.16 (s, C-Boc), 80.22 (s, C-*t*Bu), 57.42 (d, Cα-Val), 44.50 (t, Cα-Gly), 31.58 (d, Cβ-Val), 28.46 and 28.20 (q, CH_3_-Boc, CH_3_-*t*Bu), 19.08 (q, Cγ-Val), 17.68 (q, Cγ’-Val); FAB^+^MS *m/z*: 331 (28) [M + H]^+^, 275 (18) [M + H - C_4_H_8_]^+^, 219 (100) [M + H - C_4_H_8_ - C_4_H_8_]^+^, 175 (20) [M + H - C_4_H_8_ - C_5_H_8_O_2_]^+^, 72 (17) [C_4_H_8_O]^+^, 57 (20) [C_4_H_9_]^+^; HRFAB^+^MS: observed 331.2251 [M + H]^+^, (calcd. for C_16_H_31_N_2_O_5_, 331.2233).

*Boc-Phe-Val-OtBu* (**2i**): White solid; Mp 119-121 °C; [α]_D_^Hg^ (365 nm) + 9.0 (c 0.5, CHCl_3_); IR: 3,327, 2,975, 2,933, 1,735, 1,687, 1,651, 1,538, 1,367, 1,252, 1,165, 1,025, 855 cm^-1^; ^1^H-NMR (CDCl_3_) δ 7.32 - 7.10 (5H, Ar), 6.56 (1H, d, *J* = 8.4 Hz, NH-Phe), 5.20 (1H, d, *J* = 8.0 Hz, NH-Val), 4.41 (1H, m, Hα-Phe), 4.36 (1H, dd, *J* = 8.0, 4.8 Hz, Hα-Val), 3.10 (2H, dd, *J* = 13.6, 6.4 Hz, Hβ-Phe), 3.04 (1H, dd, *J* = 13.6, 6.8 Hz, Hβ’-Phe), 2.17 (1H, hd, *J* = 6.8, 4.8 Hβ-Val), 1.45 (9H, s, CH_3_Boc), 1.41 (9H, s, CH_3_*t*Bu), 0.88 (3H, d, *J* = 6.8 Hz, Hγ-Val), 0.89 (3H, d, *J* = 6.8 Hz, Hγ’-Val); ^13^C-NMR (CDCl_3_) δ 171.07 (s, CO-Phe), 170.45 (s, CO-Val), 155.39 (s, CO-Boc), 136.69 (s, C_1_), 129.36 (d, C_2_ and C_6_), 128.58 (d, C_3_ and C_5_), 126.85 (d, C_4_), 81.98 (s, C-Boc), 80.10 (s, C-*t*Bu), 57.65 (d, Cα-Val), 55.95 (d, Cα-Phe), 38.23 (t, Cβ-Phe), 31.62 (d, Cβ-Val), 28.43 and 28.26 (q, CH_3_-Boc, CH_3_-*t*Bu), 18.92 (q, Cγ-Val), 17.91 (q, Cγ’-Val); FAB^+^MS *m/z*: 421 (46) [M + H]^+^, 365 (15) [M + H - C_4_H_8_]^+^, 309 (100) [M + H - C_4_H_8_ - C_4_H_8_]^+^, 265 (96) [M + H - C_4_H_8_ - C_5_H_8_O_2_]^+^, 120 (40), 72 (32) [C_4_H_8_O]^+^, 57 (44) [C_4_H_9_]^+^; HRFAB^+^MS: observed 421.2688 [M + H]^+^, (calcd. for C_23_H_37_N_2_O_5_, 421.2702).

*Boc-Val-Val-OtBu* (**2j**): White solid; Mp 132-134 °C; [α] - 6.8 (c 1.1, CHCl_3_); IR: 3,309, 2,972, 2,934, 2,888, 1,744, 1,685, 1,651, 1,536, 1,464, 1,372, 1,301, 1,254, 1,219, 1,157, 1,017, 855 cm^-1^; ^1^H-NMR (CDCl_3_) δ 6.35 (1H, d, *J* = 7.6 Hz, NH-Val), 5.09 (1H, d, *J* = 8.8 Hz, NH-Val), 4.36 (1H, dd, *J* = 8.4, 4.4 Hz, Hα-Val), 3.86 (1H, dd, *J* = 7.6, 7.6 Hz, Hα-Val), 2.08 (2H, m, Hβ-Val, Hβ-Val), 1.40 (9H, s, CH_3_Boc), 1.38 (9H, s, CH_3_*t*Bu), 0.90, 0.87, 0.86, 0.84 (3H each, d, *J* = 6.8 Hz, Hγ-Val, Hγ’-Val, Hγ-Val, Hγ’-Val); ^13^C-NMR (CDCl_3_) δ 171.58 (s, CO-Val), 170.81 (s, CO-Val), 155.93 (s, CO-Boc), 81.16 (s, C-Boc), 80.00 (s, C-*t*Bu), 60.38 (d, Cα-Val), 57.70 (d, Cα-Val), 31.64 (d, Cβ-Val), 31.06 (d, Cβ-Val), 28.56 and 28.29 (q, CH_3_-Boc, CH_3_-*t*Bu), 19.57, 19.16, 18.21, 17.96 (q, Cγ-Val, Cγ’-Val, Cγ-Val, Cγ’-Val); FAB^+^MS *m/z*: 373 (46) [M + H]^+^, 317 (28) [M + H - C_4_H_8_]^+^, 261 (89) [M + H - C_4_H_8_ - C_4_H_8_]^+^, 217 (94) [M + H - C_4_H_8_ - C_5_H_8_O_2_]^+^, 116 (26) [C_5_H_10_NO_2_]^+^, 72 (100) [C_4_H_8_O]^+^, 57 (47) [C_4_H_9_]^+^; HRFAB^+^MS: observed 373.2711 [M + H]^+^, (calcd. for C_19_H_37_N_2_O_5_, 373.2702).

*Boc-Phe-Sar-OtBu* (**2k**): Colorless syrup (73:27 rotamer mixture); [α] - 22.5 (c 1.58, CHCl_3_); IR: 3,427, 3,322, 2,978, 2,933, 1,741, 1,710, 1,652, 1,491, 1,367, 1,236, 1,164, 1,049, 1,020, 952, 850, 759 and 701 cm^-1^; ^1^H-NMR (CDCl_3_) δ 7.30-7.00 (5H, m, Ar), 5.31 (1H, d, *J* = 8.8 Hz, NH-Phe), 4.80 (1H, dd, *J* = 15.4, 6.6 Hz, Hα-Phe), 3.92 (1H, d, *J* = 17.2 Hz, Hα-Sar), 3.84 (1H, d, *J* = 17.2 Hz, Hα’-Sar), 2.83 (3H, s, NCH_3_-Sar), 2.97 (1H, dd, *J* = 13.6, 7.2 Hz, Hβ-Phe), 2.90-2.86 (1H, m, Hβ’-Phe), 1.38 (9H, s, CH_3_-Boc), 1.31 (9H, s, CH_3_-*t*Bu); ^13^C-NMR (CDCl_3_) δ 172.11 (s, CO-Phe), 167.89 (s, CO-Sar), 155.09 (s, CO-Boc), 136.42 (s, C_1_), 129.65 (d, C_2_,C_6_), 128.38 (d, C_3_,C_5_), 126.85 (d, C_4_), 82.03 (s, C-*t*Bu), 79.67 (s, C-Boc), 51.52 (d, Cα-Phe), 50.45 (t, Cα-Sar), 39.57 (t, Cβ-Phe), 36.34 (q, NCH_3_-Sar), 28.49 and 28.24 (q, CH_3_-Boc, CH_3_-*t*Bu); FAB^+^MS *m/z*: 393 (27) [M + H]^+^, 337 (15) [M + H - C_4_H_8_]^+^, 281 (76) [M + H - 2 C_4_H_8_]^+^, 263 (15) [M + H - C_4_H_8_ - C_4_H_8_O]^+^, 237 (100) [M + H - C_4_H_8_ - C_5_H_8_O_2_]^+^, 164 (18), 120 (81), 90 (43) [C_7_H_6_]^+^, 57 (78) [C_4_H_9_]^+^; HRFAB^+^MS: observed 393.2409 [M + H]^+^, (calcd. for C_21_H_33_N_2_O_5_, 393.2389).

*Boc-Phe-Sar-OMe* (**2l**): Colorless syrup (77:23 rotamer mixture); [α] + 19.1 (c 0.54, CHCl_3_); IR: 3,427, 3,322, 2,977, 2,943, 1,751, 1,708, 1,652, 1,490, 1,407, 1,365, 1,250, 1,212, 1,171, 1,047, 1,021, 751 and 702 cm^-1^; ^1^H-NMR (CDCl_3_) δ 7.32-7.18 (5H, m, Ar), 5.34 (1H, d, *J* = 8.8 Hz, NH-Phe), 4.88 (1H, dd, *J* = 14.8, 6.8 Hz, Hα-Phe), 4.14 (1H, d, *J* = 17.2 Hz, Hα-Sar), 3.99 (1H, d, *J* = 17.2 Hz, Hα'-Sar), 3.73 (3H, s, OCH_3_), 3.04 (1H, dd, *J* = 13.6, 7.2 Hz, Hβ-Phe), 2.95 (1H, dd, *J* = 13.6, 6.4 Hz, Hβ’-Phe), 2.87 (3H, s, NCH_3_-Sar), 1.40 (9H, s, CH_3_-Boc); ^13^C-NMR (CDCl_3_) δ 172.35 (s, CO-Phe), 169.28 (s, CO-Sar), 155.07 (s, CO-Boc), 136.33 (s, Ar), 129.65 (d, Ar), 128.45 (d, Ar), 126.93 (d, C_4_), 79.81 (s, C-Boc), 52.38 (q, OCH_3_), 51.59 (d, Cα-Phe), 49.66 (t, Cα-Sar), 39.80 (t, Cβ-Phe), 36.43 (q, NCH_3_-Sar), 28.55 (q, CH_3_-Boc); FAB^+^MS *m/z*: 351 (33) [M + H]^+^, 295 (55) [M + H - C_4_H_8_]^+^, 251 (100) [M + H - C_5_H_9_O_2_]^+^, 164 (16), 120 (67), 104 (70) [C_4_H_10_NO_2_]^+^, 57 (44) [C_4_H_9_]^+^, 44 (18); HRFAB^+^MS: observed 351.1907 [M + H]^+^, (calcd. for C_18_H_27_N_2_O_5_, 351.1920).

### 3.4. General procedure for the syntheses of 2,5-diketopiperazines ***3a-3k***

Each *Nα*-Boc-dipeptidyl ester (0.25 mmol) was dissolved or suspended in water (1 mL) and heated during 10 minutes at 250 °C and 150 psi, using a monomode CEM Discover microwave apparatus at 250 W. The resulting suspension was filtered through a Hirsch funnel and washed with water (5 mL), the solid was dried under high vacuum and analyzed without further purification by NMR. Compounds **3h** and **3k** were water soluble, and in these cases, resulting solutions were lyophilized and the solids purified as indicated in Table 2 and analyzed by NMR. 

*Cyclo[Val-Orn(Cbz)]* (**3a**): White solid; Mp 202-204 °C {lit [33] 206-208 °C}; [α] - 26.0 (c 0.27, DMSO) {lit [33] - 47.4 (c 1 %)}; IR: 3,434, 3,333, 3,201, 3,094, 3,052, 2,965, 2,878, 1,678, 1,531, 1,444, 1,258, 1,142, 1,025, 774, 696, 629 cm^-1^; ^1^H-NMR (DMSO): δ 8.14 (1H, s, NH-Orn), 8.04 (1H, s, NH-Val), 7.39-7.26 (6H, m, Ar, NHδ-Orn), 5.00 (2H, s, CH_2_-Cbz), 3.81 (1H, t, *J* = 4.8 Hz, Hα-Orn), 3.66 (1H, bs, Hα-Val), 2.97 (1H, dd, *J* = 12.8, 6.4 Hz, Hδ-Orn), 2.14 (1H, m, Hβ-Val), 1.69 (1H, m, Hβ-Orn), 1.62 (1H, m, Hβ’-Orn), 1.46 (2H, m, Hγ-Orn), 0.93 (3H, d, *J* = 7.2 Hz, Hγ-Val), 0.82 (3H, m, d, *J* = 7.2 Hz, Hγ’-Val); ^13^C-NMR (DMSO): δ 167.83 (s, CO-Orn), 166.88 (s, CO-Val), 156.06 (s, CO-Cbz), 137.21 (s, Ar), 128.33 (d, Ar), 127.64 (d, Ar), 65.18 (t, CH_2_-Cbz), 59.43 (d, Cα-Val), 53.75 (d, Cα-Orn), 40.18 (t, Cδ-Orn), 31.33 (d, Cβ-Val), 31.16 (t, Cβ-Orn), 25.35 (t, Cγ-Orn), 18.74 (q, Cγ-Val), 17.29 (q, Cγ’-Val); FAB^+^MS *m/z*: 348 (87) [M + H]^+^, 307 (100) [M + H - C_3_H_6_]^+^, 289 (61), 240 (22) [M + H - C_7_H_8_O]^+^, 219 (25) [C_12_H_15_N_2_O_2_]^+^, 214 (12), 195 (12), 165 (12); HRFAB^+^MS: observed 348.1887 [M + H]^+^, (calcd. for C_18_H_26_N_3_O_4_, 348.1923).

*Cyclo[Val-D-Orn(Cbz)]* (**3b**): White solid; Mp 212-214 °C;[α] + 11.7 (c 0.5, MeOH);IR: 3,347, 3,192, 3,054, 2,962, 1,675, 1,540, 1,460, 1,265, 1,143, 1,031, 852, 696 cm^-1^; ^1^H-NMR (DMSO): δ 8.11 (1H, s, NH-Orn), 7.32 (6H, m, Ar, NH-Val), 4.99 (2H, s, CH_2_-Cbz), 4.03 (1H, bs, Hα-Val), 3.88 (1H, bt, Hα-Orn), 2.96 (1H, d, *J* = 5.6 Hz, Hδ-Orn), 2.09 (1H, m, Hβ-Val), 1.65 (1H, m, Hβ-Orn), 1.62 (1H, m, Hβ’-Orn), 1.41 (2H, m, Hγ-Orn), 0.92 (3H, d, *J* = 6.8 Hz, Hγ-Val), 0.83 (3H, m, d, *J* = 7.0 Hz, Hγ’-Val); ^13^C-NMR (DMSO): δ 168.13 (s, CO-Orn), 167.67 (s, CO-Val), 156.17 (s, CO-Cbz), 137.25 (s, Ar), 128.44 (d, Ar), 127.77 (d, Ar), 65.30 (t, CH_2_-Cbz), 59.79 (d, Cα-Val), 53.27 (d, Cα-Orn), 40.18 (t, Cδ-Orn), 32.19 (d, Cβ-Val), 29.64 (t, Cβ-Orn), 24.41 (t, Cγ-Orn), 18.54 (q, Cγ-Val), 17.08 (q, Cγ’-Val); FAB^+^MS *m/z*: 348 (3) [M + H]^+^, 219 (5), 154 (12), 130 (30), 107 (8), 91 (100), 85 (28), 72 (25); HRFAB^+^MS: observed 348.1949 [M + H]^+^, (calcd. for C_18_H_26_N_3_O_4_, 348.1923).

*Cyclo[Phe-Orn(Cbz)]* (**3c**): White solid; Mp 200-202 °C {lit [33] 210-212 °C}; [α] - 24.8 (c 0.24, DMSO) {lit [33] - 12.9 (c 1 %)}; IR: 3,322, 3,189, 3,039, 2,965, 2,894, 1,669, 1,532, 1,458, 1,338, 1,246, 1,134, 1,101, 1,016, 852, 753, 669 cm^-1^; ^1^H-NMR (DMSO): δ 8.16 (1H, s, NH-Phe), 8.05 (1H, s, NH-Orn), 7.40 - 7.05 (10H, m, Ar), 5.00 (2H, s, CH_2_-Cbz), 4.17 (1H, s, Hα-Phe), 3.57 (1H, bs, Hα- Orn), 3.13 (1H, dd, *J* = 13.6, 4.0 Hz, Hβ-Phe), 2.83 (1H, dd, *J* = 13.6, 5.2 Hz, Hβ’-Phe), 2.68 (1H, dd, *J* = 12.8, 6.4 Hz, Hδ-Orn), 1.04 (1H, m, Hβ-Orn), 0.85 (2H, m, Hγ-Orn), 0.65 (2H, m, Hβ’-Orn); ^13^C-NMR (DMSO): δ 166.70 (s, CO-Orn), 166.00 (s, CO-Phe), 155.79 (s, CO-Cbz), 137.16 (s, Ar-Cbz), 135.92 (s, Ar), 130.16 (d, Ar), 128.27 (d, Ar), 127.92 (d, Ar), 127.69 (d, Ar), 126.57 (d, Ar), 65.10 (t, CH_2_-Cbz), 55.30 (d, Cα-Phe), 53.61 (d, Cα-Orn), 39.95 (t, Cδ-Orn), 38.16 (t, Cβ-Phe), 30.66 (t, Cβ-Orn), 24.33 (t, Cγ-Orn); FAB^+^MS *m/z*: 396 (100) [M + H]^+^, 352 (32) [M + H - H_2_NCO]^+^, 335 (9), 307 (53) [M + H - C_7_H_6_]^+^, 289 (32) [M + H - C_7_H_7_O]^+^, 243 (16) [M + H - C_14_H_16_NO_2_]^+^, 219 (18) [M + H - C_10_H_12_NO_2_]^+^, 165 (14); HRFAB^+^MS: observed 396.1949 [M + H]^+^, (calcd. for C_22_H_26_N_3_O_4_, 396.1923).

*Cyclo(Phe-Gly)* (**3e**): White solid; Mp 266-268 °C {lit [34] 271-273 °C }; [α] + 26.6 (c 0.95, DMSO) {lit [34] + 7.3 (c 0.95, DMSO)}; IR: 3,426, 3,190, 3,057, 2,977, 2,921, 2,878, 1,676, 1,462, 1,332, 1,086, 1,004, 847, 794, 758, 702 cm^-1^; ^1^H- and ^13^CNMR (DMSO) are in agreement with previously reported data [35]; FAB^+^MS *m/z*: 205 (13) [M + H]^+^, 169 (15), 154 (24), 130 (43) [M + H - C_4_H_4_]^+^, 85 (100) [C_3_H_3_NO_2_]^+^; HRFAB^+^MS: observed 205.1058 [M + H]^+^, (calcd. for C_11_H_13_N_2_O_2_, 205.0977).

*Cyclo(Phe-Phe)* (**3f**): White solid; [α] - 42.8 (c 0.2, AcOH); ^1^H- and ^13^C-NMR (DMSO), and HRFAB^+^MS are in agreement with previously reported data [36]; IR: 3,440, 3,318, 3,198, 3,056, 2,970, 2,929, 1,669, 1,458, 1,338, 1,197, 1,088, 1,013, 759 and 700 cm^-1^.

*Cyclo(Val-Phe)* (**3g**): White solid; Mp 264-266 °C {lit [36] 263-265 °C}; [α] - 66.0 (c 0.28, DMSO) {lit [37] - 64 (c 0.2 AcOH); [34] - 43.3 (c 0.27 DMSO)}; IR: 3,440, 3,316, 3,192, 3,056, 2,967, 2,885, 1,668, 1,454, 1,341, 1,090, 859, 758, 699, cm^-1^; ^1^H- and ^13^C-NMR (DMSO) are in agreement with previously reported data [34,37]; FAB^+^MS *m/z*: 247 (100) [M + H]^+^, 219 (13) [M + H - CO]^+^, 217 (10), 203 (7), 165 (5); HRFAB^+^MS: observed 247.1446 [M + H]^+^, (calcd. for C_14_H_19_N_2_O_2_, 247.1447).

*Cyclo(Val-Gly)* (**3h**): White solid; Mp 210-212 °C {lit [38] 256 °C}; [α] + 32.9 (c 0.46, H_2_O) {lit [38] + 23.7 (c 1.0, H_2_O)}; IR: 3,431, 3,199, 3,055, 2,925, 2,859, 1,670, 1,461, 1,381, 1,346, 1,109, 1,047, 808, 621 cm^-1^; ^1^H- and ^13^C-NMR (DMSO) are in agreement with previously reported data [38,39]; FAB^+^MS *m/z*: 157 (94) [M + H]^+^, 154 (100) [M - H_2_], 136 (78), 120 (12), 107 (25), 85 (58) [C_5_H_9_O]^+^, 77 (25), 55 (32), 43 (27) [C_3_H_7_]^+^, 41 (25); HRFAB^+^MS: observed 157.0957 [M + H]^+^, (calcd. for C_7_H_13_N_2_O_2_, 157.0957).

*Cyclo(Val-Val)* (**3j**): Colorless needless; Mp 268 °C with sublimation {lit [40] 268 °C}; [α] - 54.8 (c 0.5, AcOH) {lit [15] - 62 (c 0.5, AcOH); IR: 3,434, 3,325, 3,193, 3,100, 3,057, 2,966, 2,880, 1,664, 1,449, 1,346, 1,293, 847 cm^-1^; ^1^H-NMR (DMSO): δ 7.96 (1H, s, NH-Val), 3.69 (1H, s, Hα-Val), 2.18 (1H, m, Hβ-Val), 0.95 (3H, d, *J* = 7.6 Hz, Hγ-Val), 0.83 (3H, d, *J* = 7.6 Hz, Hγ’-Val); ^13^C-NMR (DMSO): δ 167.26 (s, CO), 59.09 (d, Cα), 31.06 (d, Cβ), 18.73 (q, Cγ), 17.33 (q, Cγ’); FAB^+^MS *m/z*: 199 (100) [M + H]^+^, 197 (19), 169 (8); HRFAB^+^MS: observed 199.1463 [M + H]^+^, (calcd. for C_10_H_19_N_2_O_2_, 199.1447).

*Cyclo(Sar-Phe)* (**3k**): Colorless needles; Mp 180-183 °C {lit [41] 184-185 °C}; [α] + 56.0 (c 1.01, MeOH) {lit [41] + 47.5 (c 2.3 MeOH)}; IR: 3,440, 3,253, 2,963, 2,929, 2,895, 1,656, 1,470, 1,441, 1,322, 1,102, 1,033, 870, 754, 699, cm^-1^; ^1^H-NMR (CDCl_3_): δ 7.60 (1H, bs, NH-Phe), 7.35-7.16 (5H, m, Ar), 4.31 (1H, bs, Hα-Phe), 3.48 (1H, d, *J* = 17.6 Hz, Hα-Sar), 3.24 (1H, dd, *J* = 13.6, 5.2 Hz, Hβ-Phe), 3.07 (1H, dd, *J* = 13.6, 4.4 Hz, Hβ’-Phe), 2.81 (3H, s, NCH_3_-Sar), 2.79 (1H, d, *J* = 17.6 Hz, Hα’-Sar); ^13^C-NMR (CDCl_3_): δ 166.32 (s, CO-Sar), 165.52 (s, CO-Phe), 134.99 (s, Ar), 130.07 (d, Ar), 128.63 (d, Ar), 127.58 (d, C_4_), 56.53 (d, Cα-Phe), 50.92 (t, Cα-Sar), 40.94 (t, Cβ-Phe), 33.66 (s, NCH_3_-Sar); FAB^+^MS *m/z*: 219 (100) [M + H]^+^, 154 (50) [M + H - CO]^+^, 136 (56), 91 (35) [C_7_H_7_]^+^, 73 (58); HRFAB^+^MS: observed 219.1134 [M + H]^+^, (calcd. for C_12_H_15_N_2_O_2_, 219.1134).

## 4. Conclusions

Optically pure *cis*-DKPs could be synthesized in one-pot from the corresponding *Nα*-Boc-dipeptidyl-*tert*-butyl esters in water under microwave irradiation for ten minutes. Employing these conditions, the *tert*-butoxy group is efficiently removed, leading to cyclization in excellent yields. This is the first protocol for ring closures using *Nα*-Boc-dipeptidyl-*tert*-butyl esters. The *trans*-DKP fragment present in the natural product **1** was synthesized in quantitative yield. The reaction is rapid, secure, environmentally friendly and highly efficient.
